# Pregnancy Among Women Receiving Chronic Dialysis in France (2006–2020)

**DOI:** 10.1016/j.ekir.2024.05.008

**Published:** 2024-05-15

**Authors:** Hayet Baouche, Cécile Couchoud, Henri Boulanger, Salima Ahriz-Saksi, Imene Mansouri, Abdelaziz Hamani, Pierre Taupin, Xavier Ferreira, Marine Panaye, Julien Stirnemann, Olivier Moranne, Jean-Philippe Jais, REIN registry

**Affiliations:** 1 Biostatistics Department, APHP-Necker-Enfants Malades Hospital, REIN Registry, Paris, France; 2 French REIN registry, Agence de la Biomédecine, La Plaine Saint-Denis, Paris, France; 3 Nephrology and Dialysis Department, Estrée Clinic, Stains, Paris, France; 4 Dialysis Department, Jules Valles Hospital, Athis Mons, Essonne, France; 5 Medical information Department-PMSI, APHP-Necker-Enfants Malades Hospital, Paris, France; 6 Nephrology Department, Edouard-Herriot Hospital, Pavilion P5, Lyon, France; 7 Obstetrics and Maternal-Fetal Medicine Department, APHP-Necker-Enfants Malades Hospital, Paris, France; 8 Research Team EA 7328, Paris Cité University, Paris, France; 9 Nephrology, Dialysis-Apheresis Unit, Caremeau University Hospital, Nîmes, France; 10 UMR Inserm-UM, Desbrest Institute of Epidemiology and Public Health, Montpellier, France

**Keywords:** chronic kidney failure, pregnancy, pregnancy complications, pregnancy outcome, renal dialysis

## Abstract

**Introduction:**

In women receiving chronic dialysis, fertility is impaired. The objectives of this study were to estimate the incidence rate of pregnancies among women of childbearing age (15–50 years) receiving chronic dialysis from 2006 to 2020 in France, to describe the pregnancy outcomes and renal management during pregnancy.

**Methods:**

This national observational, retrospective study was based on data from the French REIN registry matched with the National Health Data System.

**Results:**

Over the period 2006 to 2020 in France, 348 pregnancies were identified in 240 women receiving chronic dialysis. The overall incidence of pregnancy was 11.1, 95% confidence interval (CI) (9.9–12.3) cases per 1000 person-years. Hemodialysis was the predominant modality during pregnancy. Main maternal complications were preeclampsia (*n* = 19) and gestational diabetes (*n* = 11). The most obstetric complications were premature rupture of membranes (*n* = 14) and polyhydramnios (*n* = 5). These pregnancies resulted in 174 (50%) abortions (<22 weeks), including 104 elective abortions (29.9%), 44 miscarriages (12.6%), 17 therapeutic abortions (4.9%), 5 ectopic pregnancies (1.4%), and 4 hydatidiform moles (1.2%). The remaining 174 (50%) pregnancies with deliveries (≥22 weeks) resulted in 166 live births (70 full-term [42.2%], 96 preterm births [57.8%]), and 8 stillbirths. Median gestational age was 36 weeks (32–38) for 174 deliveries.

**Conclusion:**

There have been improvements in maternal and fetal outcomes regarding pregnancy on chronic dialysis. However, our study shows a significant proportion of elective abortions. Better fertility management of women receiving chronic dialysis is advised by contraception or by pregnancy planning and early multidisciplinary follow-up.

## Graphical abstract

**Figure:**
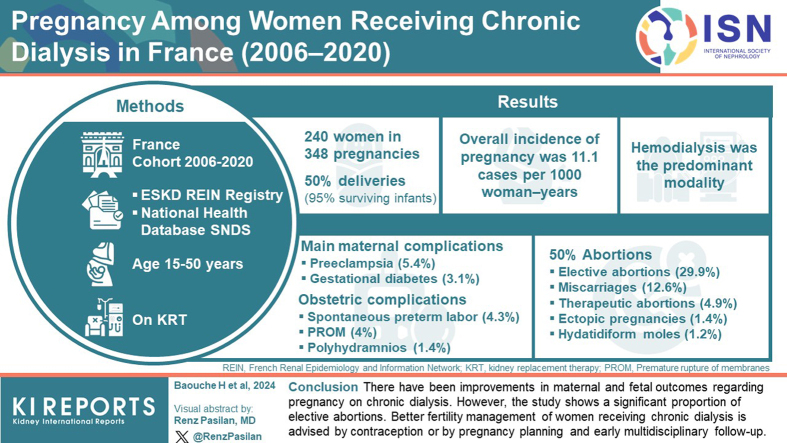

Owing to disruption of the hypothalamic-pituitary axis, fertility is impaired in women of childbearing age receiving chronic dialysis, although improvements in dialysis conditions over the past decades have increased the fertility in these patients.^1^, ^2^, ^3^ Adequate fetal monitoring, progress in obstetrics, and neonatal care have improved the prognosis of pregnancies in women on chronic dialysis. However, significant obstetric/maternal risks remain (preeclampsia, hemorrhagic accidents including retro placental hematoma, worsening of anemia, liver abnormalities including cholestasis of pregnancy, and the risk of anti-HLA immunization of the mother, which would compromise a future transplant) as well as risks for the fetus (prematurity, intrauterine growth retardation, chronic hypoxia in the event of maternal anemia, and fetal death in utero).^4^, ^5^, ^6^, ^7^ Therefore, pregnancy remains a rare and challenging event in women receiving chronic dialysis. According to the literature, the frequency of conception varies between countries (1–7% of women), and the live birth rate varies according to series from 50% to 100%.^2^^,^^4^^,^^5^^,^^8^^,^^9^

In France, the exact pregnancy rate among women receiving chronic dialysis with end-stage kidney disease is unknown, because there is no systematic national census to continuously assess the evolution of the incidence, the prognosis of these pregnancies, and the impact of changes in medical practices. The only few recent data available on maternal-fetal outcomes and complications of these pregnancies are surveys of the centers, but they are not exhaustive.^10^, ^11^, ^12^

The primary objective of this study was to estimate the trend in incidence rates of pregnancies in France among women of childbearing age (15–50 years) receiving chronic dialysis over the period from 2006 to 2020. The secondary objectives were to describe pregnancy outcomes (rate of live births, gestational age, rate of miscarriages, and abortions) and renal management during pregnancy.

## Methods

This is an observational, retrospective study using 2 national databases.

### Data Sources

The REIN registry (Epidemiology and Information Network in Nephrology) is a national register whose institutional support is the Biomedicine Agency. Its objective is the epidemiological follow-up of all patients with end-stage kidney disease treated by kidney replacement therapy in France.^13^^,^^14^ The REIN started in 2002 and was deployed progressively region by region reaching complete national coverage (France and overseas) in 2012; clinical, demographic, and laboratory data are collected at the start of kidney replacement therapy along with dialysis modalities and are updated annually. Events such as death, transfer, withdrawal from dialysis, placement on a transplant waiting list, and kidney transplantation (from living or deceased donors) are systematically reported in real time.

The SNDS (National Health Data System) contains detailed information on medical care consumption for almost the entire population of France. It includes the DCIR (Inter-Plan Consumption Data), which contains individualized and exhaustive information on reimbursements of Health Insurance beneficiaries related to their consumption of ambulatory care and the date of delivery of these services. It also includes the PMSI (Information Systems Medicalization Program), an exhaustive public and private hospital discharge database containing, in particular, all patient characteristics, such as age, gender, town of residence, duration of hospitalization, diagnoses coded according to the International Classification of Diseases-10: main diagnosis, related diagnosis, associated diagnoses and medical acts.^15^

Data from the REIN registry were combined with data from the SNDS via deterministic indirect matching. This consists of identifying patients who are present in both databases by the comparison of the shared information available in the 2 databases: age, sex, town of residence, mo/yr of the first transplant or first dialysis session, the center where the first kidney replacement therapy care took place and the month/year of death, if relevant.^16^

### Study Population

This study included all women of childbearing age (15–50 years) residing in metropolitan France, French overseas departments, as well as patients from New Caledonia and French Polynesia identified during their care in Metropolitan France (fallback), who became pregnant between January 1, 2006, and December 31, 2020 (SNDS information) recorded in the REIN registry as being treated by dialysis. Women who had conceived before chronic dialysis and who started dialysis during pregnancy were also included. All pregnancies were included whatever the outcome: childbirth or any other outcome (ectopic pregnancy, spontaneous miscarriage, elective abortions, therapeutic abortions, and premature or full-term delivery). According to French law, pregnancy can be terminated at the mother’s request at any gestational age in the event of a severe and incurable disorder. It also permits termination <16 weeks without any particular medical reason, based only on parental decision.

### Available Information

The required variables available in the REIN registry were demographic data, age at start of dialysis, primary renal disease, comorbidities, type of dialysis (hemodialysis [HD] or peritoneal dialysis [PD]) as well as the amount of HD before pregnancy, vascular access, laboratory results (hemoglobin and serum albumin levels), registration on the waiting list for the transplant and the long-term maternal outcome (renal transplantation and death).

The data retrieved from the SNDS were the modality of dialysis and the number of HD sessions during pregnancy; maternal and obstetric complications during pregnancy; fetal and neonatal complications; pregnancy outcome; gestational age at the end of the pregnancy, and mode of delivery. The International Classification of Diseases-10) codes used to define these outcomes are listed in Supplementary Table S1. Their characteristics were studied according to the number of medical acts and diagnoses, including the main, associated, and related diagnoses.

### Identification and Dating of Pregnancies

Given that the REIN register does not collect data on pregnancies in women on chronic dialysis, pregnancies were identified via the hospital discharge database according to their outcomes. Our algorithm identified hospital stays which a diagnosis related to pregnancy, miscarriage, elective or therapeutic termination, or childbirth (Supplementary Table S1). The onset of pregnancy was estimated based on the pregnancy end date and gestational age or time since the last menstrual period as available in the hospital discharge database. Miscarriages and therapeutic abortions treated by a general practitioner were not identified.

### Statistical Analyses

Standard descriptive statistics were used for all variables of interest. Normally distributed quantitative variables were expressed as mean and SD; nonnormally distributed variables were expressed as median and interquartile range. Qualitative variables were summarized by the frequencies and the percentages.

The rate of dialyzed women with pregnancy was calculated by taking as the denominator the number of all women of childbearing age (15–50 years) receiving chronic dialysis over the same period and taking into account the gradual integration of the regions in the REIN registry over time.

The incidence rate was calculated by relating the number of new cases of pregnancy to the size of the population of women of childbearing age on chronic dialysis during the same period. Rates were expressed as the number of pregnancies per thousand person-years (the summed duration of follow-up of women in the population of dialysis patients of childbearing age). Because REIN and the SNDS were not exhaustive before 2008, incidence rates were calculated only for the 2008 to 2020 period.

Nephrological management during pregnancy according to pregnancy stage and the woman’s trajectory were analyzed per trimester and represented in a Sankey diagram. Because women may experience several pregnancies, we descriptively compared the outcome of pregnancy according to maternal and renal risk factors in terms of abortions (<22 weeks) versus deliveries (≥22 weeks), and preterm deliveries (22–36 weeks) versus full-term deliveries (≥37 weeks). We used the following variables: age at conception, parity, cause of end-stage kidney disease, at least 1 comorbidity declared before pregnancy, obesity, active smoking, activity, pregnancy period, timing of conception (conception on dialysis [COD], conception before dialysis [CBD]), dialysis method (HD, PD), and mode (conventional dialysis and daily dialysis) during trimesters.

Following the World Health Organization definitions and international statistical classification of diseases and related health problems, live birth was calculated on the entire cohort, irrespective of the duration of the pregnancy, as in other study on the subject.^2^^,^^5^

Women were monitored up to December 30, 2020. All data were analyzed using R statistical software, version 4.1.2 (R Core Team, Vienna, Austria, www.R-project.org).

## Results

In total, during the period from 2006 to 2020 in France, 348 pregnancies were identified in 240 women of childbearing age (15–50 years) receiving chronic dialysis (Supplementary Figure S1), and 166 surviving infants.

Supplementary Figure S2 shows the geographical distribution of these 348 pregnancies and their outcomes in mainland France and overseas departments and territories. The overall incidence rate of pregnancy on dialysis was 11.1, 95% CI: (9.9–12.3) cases per thousand person-years, ranging from 5.4, 95% CI: (2.6–9.6) for the year from 2008 to 10.9, 95% CI: (7.2–15.9) in 2020. The overall frequency of conception was 0.46% (341/74269), ranging from 0.22% in 2008 to 0.48% in 2020 (Table 1).

**Table 1:** Frequency of chronic dialysis pregnancies and crude incidence rate of pregnancies per 1000 Person-years between 2008 and 2020

Year	*n*	*N*	%	Person–yr	Incidence rate	95% CI	Number of regions included
2008	11	4852	0.22%	2036.54	5.40	2.69–9.66	21
2009	28	5120	0.54%	2114.28	13.24	8.8–19.14	22
2010	15	5254	0.28%	2151.63	6.97	3.90–11.49	24
2011	24	5558	0.43%	2286.78	10.49	6.72–15.61	26
2012	31	5728	0.54%	2385.04	12.99	8.83–18.44	27
2013	30	5976	0.50%	2436.07	12.31	8.31–17.58	27
2014	25	6022	0.41%	2493.01	10.03	6.49–14.80	27
2015	30	5971	0.50%	2486.53	12.06	8.14–17.22	27
2016	33	6024	0.54%	2429.89	13.58	9.35–19.07	27
2017	33	6008	0.55%	2431.54	13.57	9.34–19.06	27
2018	27	6043	0.44%	2474.59	10.91	7.19–15.87	27
2019	27	6090	0.44%	2466.08	10.95	7.21–15.93	27
2020	27	5623	0.48%	2459.41	10.97	7.23–15.97	27
Total	341	74269	0.46%	30651.39	11.12	9.97–12.37	27

CI, confidence interval; *N*, number of women of childbearing age on chronic dialysis; *n*, number of pregnancies.

Results for 2006 and 2007 are not shown in the table because neither the REIN register nor the SNDS can be considered exhaustive and representative at that time.

### Women’s Characteristics

Table 2 shows the characteristics of the 240 pregnant women receiving chronic dialysis. The average age at the start of dialysis was 29.8 ± 6.4 years. The primary renal diagnosis was glomerulonephritis 64 (26.6%). Forty-six women (19.2%) had declared at least 1 comorbidity, the most common being diabetes (*n* = 29; 12.1%) and lupus (*n* = 22; 9.1%). Obesity was observed in 31 women (12.9%), active smoking in 29 women (12.1%), and only 62 (25.8%) of women were active. Fifty-six women (23.3%) CBD and started dialysis treatment during pregnancy. Among the 184 women who had COD, 178 (96.7%) were on HD and 6 (3.3%) on PD before pregnancy, with a median time from the start of dialysis to pregnancy 2.5 ranging from 1.3 to 3 years. The most HD locations were in-center HD and assisted self-care HD. Of these 85.4% women had an arteriovenous fistula, and their median weekly dialysis duration was 12 hours, ranging from 7 to 18 hours. The mean hemoglobin was 10.3 ± 1.6 g/l, and the median albuminemia was 38 ranging from 13 to 47 g/l.

**Table 2:** Characteristics of women with ESKD at the first pregnancy on chronic dialysis

*N* = 240	*N* (%)
Age at the start of dialysis (yr), mean ± SD	29.8 ± 6.45
Cause of ESKD:	
Glomerulonephritis (GN)	64 (26.6%)
Hypertensive nephropathy/vascular	31 (13%)
Diabetic nephropathy (DN)	22 (9.1%)
Lupus nephritis (SLE)	19 (8%)
Pyelonephritis (PN)	13 (5.4%)
Polycystic kidney disease (PKD)	5 (2.1%)
Other causes	49 (20.4%)
Unknown	37 (15.4%)
Comorbidities and risk factors:	
At least one declared comorbidity, including:	46 (19.2%)
Diabetes	29 (12.1%)
Lupus	22 (9.1%)
Liver disease (HBV, HCV, cirrhosis)	11 (4.6%)
HIV/AIDS	7 (2.9%)
BMI (kg/m^2^)	
<18.5	24 (10%)
18.5–24	112 (46.7%)
25–29	42 (17.5%)
≥30	31 (12.9%)
Unknown	31 (12.9%)
Smoking status:	
Non-smoker	172 (71.7%)
Active smoking	29 (12.1%)
Ex-smoker	13 (5.4%)
Unknown	26 (10.8%)
Activity:	
Active	62 (25.8%)
Stay-at-home woman	24 (10%)
Unemployed	6 (2.5%)
Inactive (long sick leave and other)	61 (25.4%)
Unknown	87 (36.3%)
In the subgroup of women with conception on dialysis (COD)	
Method of dialysis before pregnancy (*N* = 184)	
Hemodialysis	178 (96.7%)
Peritoneal dialysis	6 (3.3%)
Dialysis location before pregnancy (*N* = 184)	
In-center HD	87 (47.3%)
Medicalized dialysis units	20 (10.9%)
Training center HD	1 (0.5%)
Self-Care HD	1 (0.5%)
Assisted Self-Care HD	66 (35.9%)
Home treatment	9 (4.9%)
Vascular Access before pregnancy in HD (*N* = 178)	
Arteriovenous (AV) fistula	152 (85.4%)
Central venous catheter (CVC)	23 (12.9%)
Arteriovenous (AV) graft	1 (0.6%)
Other	2 (1.1%)
Weekly dialysis dose before pregnancy in HD (*N* = 178)	
Number of hemodialysis sessions, median (IQR)	3 (3–3)
Duration of hemodialysis session (minutes), median (IQR)	4 (4–4)
Weekly dialysis dose, median (IQR)	12 (12–12)
Erythropoietin before pregnancy (*N* = 184)	
Yes	163 (92.1%)
No	12 (7.9%)
Biological data before pregnancy (*N* = 184)	
Albuminemia, g/l, mean ± SD	38 (33.9–40.5)
Hemoglobin, g/l, mean ± SD	10.3 ± 1.6

AIDS, acquired immunodeficiency syndrome; BMI, body mass index; HBV, hepatitis B virus; HCV, hepatitis C virus; HIV, human immunodeficiency virus; IQR, interquartile range; SD, standard deviation.

### Pregnancy Data and Outcomes

**Figure 1:**
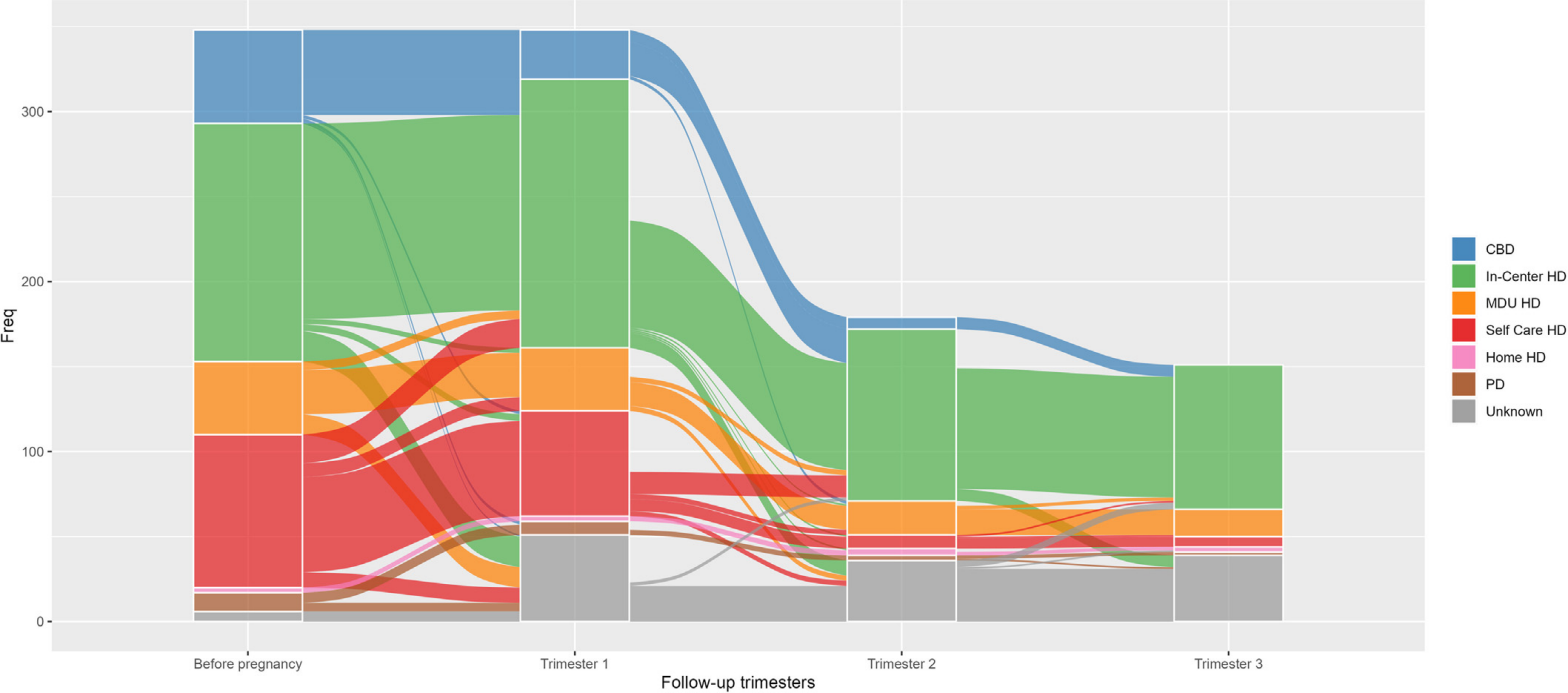
Sankey diagram representing the flow of pregnancies and dialysis modalities by trimester in France (2006-2020).

Table 3 describes maternal data for the 348 pregnancies. The median age was 32 (28–36.2) years at conception. Half of women, 178 (51.1%), were aged 25 to 34 years. Sixty-six women (27.5%) on chronic dialysis had at least 2 pregnancies during the study period. There were 292 (83.9%) pregnancies in women with conception on dialysis with conception and 56 (16.1%) pregnancies in women with CBD. The median time from the start of dialysis to pregnancy (COD) was 2.5 years, ranging from 1.3 to 3 years, and 149 out of 348 women were registered on the waiting list for a kidney transplant at the time of conception (42.8%). Table 3 and the Sankey diagram (Figure 1) show that in-center HD was the predominant modality for women’s renal replacement therapy during pregnancy. There was an increase in daily HD over time: 83 (26%), 102 (59.3%), and 83 (55%) in the first, second, and third trimesters, respectively.

**Table 3:** Maternal, obstetrical, fetal, and neonatal data on chronic dialysis pregnancies

Maternal data (*N* = 348)	*N* (%), median (IQR)
Age at conception (yr)	32 (28–36.2)
Number of pregnancies per woman on dialysis (*N* = 240)	
Pregnancy = 1	174 (72.5%)
Pregnancies ≥ 2	66 (27.5%)
Multiple pregnancies (twin) (*N* = 348)	5 (1.4%)
Timing of conception (*N* = 348):	
Conception on dialysis (COD)	292 (83.9%)
Conception before starting dialysis (CBD)	56 (16.1%)
Time from start of dialysis to pregnancy (COD) (yr)	2.5 (1.3–3)
Registration on the waiting list at conception (*N* = 348):	149 (42.8%)
Conception on dialysis (COD) (*N* = 292)	141 (48.3%)
Conception before starting dialysis (CBD) (*N* = 56)	8 (14.3%)
Residence location (*N* = 348):	
Metropolitan France	296 (85.1%)
Overseas territories	52 (14.9%)
Dialysis data during pregnancy	
Dialysis method during pregnancy:	
Trimester 1 (*N* = 319)^a^	N = 319
Hemodialysis (HD)	260 (81.5%)
In-center HD	155 (48.6%)
Medicalized dialysis units HD	37 (11.6%)
Self-care HD	62 (19.4%)
Training center HD	3 (0.9%)
Home HD	3 (0.9%)
Peritoneal dialysis (PD)	8 (2.5%)
Automated PD	6 (1.8%)
Continuous ambulatory PD	2 (0.6%)
Dialysis mode (*N* = 319)^b^	N = 319
Conventional dialysis	151 (47.3%)
Daily dialysis	83 (26%)
Conception before starting dialysis (CBD)	29 (8.3%)
Trimester 2 (*N* = 172)^c^	N = 172
Hemodialysis (HD):	133 (77.3%)
In-center HD	97 (56.4%)
Medicalized dialysis units HD	20 (11.6%)
Self-care HD	8 (4.6%)
Training center HD	4 (2.3%)
Home HD	4 (2.3%)
Peritoneal dialysis (PD):	3 (1.7%)
Automated PD	2 (1.1%)
Continuous ambulatory PD	1 (0.6%)
Dialysis mode (*N* = 172)^d^	N = 172
Conventional dialysis	14 (8.1%)
Daily dialysis	102 (59.3%)
Conception before starting dialysis (CBD)	7 (2%)
Trimester 3 (*N* = 151)^e^	N = 151
Hemodialysis (HD)	110 (72.9%)
In-center HD	82 (54.3%)
Medicalized dialysis units HD	16 (10.6%)
Self-care HD	6 (4%)
Training center HD	3 (2%)
Home HD	3 (2%)
Peritoneal dialysis (PD)	2 (1.3%)
Automated PD	1 (0.6%)
Continuous ambulatory PD	1 (0.6%)
Dialysis mode (*N* = 151)^f^	N = 151
Conventional dialysis	2 (1.3%)
Daily dialysis	83 (55%)
Obstetric data	N = 348
Maternal complications during pregnancy:	
Gestational hypertension	7 (2%)
Pre-eclampsia in the entire cohort (*N* = 348)	19 (5.4%)
Including pre-eclampsia ≥22 weeks (*N* = 174)	18 (10.3%)
Eclampsia	1 (0.3%)
Gestational Diabetes	11 (3.1%)
Liver disease during pregnancy	4 (1.1%)
Postpartum hemorrhage (PPH)	4 (1.1%)
Anemia	2 (0.5%)
Amniotic embolism	1 (0.3%)
Maternal death	1 (0.2%)
Obstetric complications:	
Preterm spontaneous labor (<37 wk)	15 (4.3%)
Premature rupture of membranes (PROM)	14 (4%)
Polyhydramnios	5 (1.4%)
Placental abruption	5 (1.4%)
Another hemorrhage before labor	4 (1.1%)
Pregnancy outcome	N = 348
Mode of delivery:	
Vaginal delivery in the entire cohorte (*N* = 348)	97 (27.8%)
Including vaginal delivery without elective abortions (N = 244)	97 (39.7%)
Cesarean section in the entire cohorte (*N* = 348)	76 (21.8%)
Including cesarean section without elective abortions (*N* = 244)	76 (31.1 %)
Gestational age of all pregnancies (wk)^g^	27 (9–36)
Total abortion (<22 wk) (1 twin pregnancy)	174 (50%)
Elective abortions	104 (29.9%)
Spontaneous abortions (miscarriages)	44 (12.6%)
Early miscarriages (<14 wk)	24 (6.9%)
Late miscarriages (14–21 wk) (1 twin)	20 (5.7%)
Therapeutic abortions	17 (4.9%)
Ectopic pregnancy	5 (1.4%)
Hydatidiform mole	4 (1.2%)
Total childbirth (≥22 wk) (4 twin pregnancies)	174 (50%)
Gestational age (for ≥22 wk),^h^*N* = 174	36 (32–38)
Live births including:	166 (47.7%)
Full term (1 twin), *N* = 166	70 (42.2%)
Preterm births <37 wk, *N* = 166	96 (57.8%)
Moderate to late preterm 32–<37 wk, (1 twin)	56 (58.3%)
Very preterm 28–<32 wk, (1 twin)	19 (19.8%)
Extremely preterm ≥22 to <28 wk, (1 twin)	21 (21.9%)
Stillbirth	8 (4.5%)
Fetal and neonatal complications after 22 wk:	
Fetal heart rate abnormalities	23 (6.6%)
Intrauterine growth retardation (IUGR)	16 (4.5%)
Hypoxia / fetal distress	7 (2%)

Missing data- ^a^51 (16%); ^b^85 (26.7%); ^c^36 (21%); ^d^56 (32.6%); ^e^39 (25.8%); ^f^66 (43.7%); ^g^51 (14.6%); ^h^8 (4.6%).

IQR, interquartile range; Wk, weeks.

Among the 348 pregnancies, 343 (98.5%) were singleton pregnancies and 5 (1.5%) were twin pregnancies; 174 ended with childbirth and 174 with abortion. The median gestational age was 32 weeks (28–36.2) of amenorrhea for 348 pregnancies (Table 3). The cause of the 174 abortions (<22 weeks) were elective abortions in 104 (29.9%), spontaneous miscarriages in 44 (12.6%), therapeutic abortions in 17 (4.9%), ectopic pregnancy in 5 (1.4%), and hydatidiform mole in 4 (1.2%). The 174 deliveries (≥22 weeks) ended with 166 (47.7%) live births, including 70 (42.2%) full-term and 96 (57.8%) preterm births with a median gestational age of 36 weeks (32–38) (Table 3).

For the 348 pregnancies, maternal complications included preeclampsia in 19 women (5.4%) and for pregnancies reached ≥22 weeks (*n* = 18; 10.3%), gestational diabetes in 11 women (3.1%), gestational hypertension in 7 women (2%), liver disease during pregnancy in 4 women (1.1%), postpartum hemorrhage in 4 women (1.1%) and eclampsia in 1 women (0.3%) (Table 3). Obstetric complications included spontaneous preterm labor in 15 women (4.3%), premature rupture of membranes in 14 women (4%), polyhydramnios in 5 women (1.4%), placental abruption in 5 (1.4%), and there were 4 other causes of hemorrhages before labor (1.1%). The only maternal death was caused by a *Staphylococcus aureus* sepsis in a woman with several comorbidities (lupus, hypertension, obesity, and hypercholesterolemia).

Preterm birth <37 weeks was the most common neonatal complication 96 (57.8%) (Table 3). Other fetal and neonatal complications included fetal heart rate abnormalities during labor in 23 (6.6%), intrauterine growth restriction in 16 (4.5%), hypoxia and fetal distress in 7 (2%) and stillbirths (fetal death after 22 weeks) in 8 of 174 (4.5%) (Table 3).

Table 4 presents the outcome of pregnancy according to maternal and renal risk factors as well as nephrological management during pregnancy. The average age at conception was higher in women whose pregnancies reached delivery at term. Nulliparous women (50%) had more abortions (abortion vs. deliveries), and 49% of women had more preterm deliveries (preterm deliveries vs. full-term deliveries). Obesity (BMI ≥30 kg/m^2^) was more common in women with premature deliveries. Abortions were higher among active women or those who had conceived whilst on chronic dialysis. The highest proportion of abortions was over the 2011 to 2015 period, whereas the proportion of deliveries was higher over the 2016 to 2020 period, with an increase in full-term deliveries.

**Table 4:** The outcome of pregnancy according to maternal and renal risk factors as well as nephrological management during pregnancy, 8 missing data on gestational age for deliveries (preterm and term)

Characteristics	Abortions (<22 wk) *N* = 174	Deliveries (≥22 wk) *N* = 174	Preterm deliveries (22–36 wk) *N* = 96	Term deliveries (≥37 wk) *N* = 70
	*N* (%), mean ± SD		*N* (%), mean ± SD	
Age at conception (yr)	31.2 ± 6.6	32 ± 5.8	31.8 ± 5.6	34.2 ± 6
Age classes:				
15–19 yr	9 (5.2%)	1 (0.6%)	0 (0%)	1 (1.4%)
20–24 yr	21 (12.1%)	17 (9.7%)	12 (12.5%)	4 (5.7%)
25–29 yr	32 (18.4%)	37 (21.3%)	25 (26.1%)	10 (14.3%)
30–34 yr	60 (34.5%)	49 (28.2%)	27 (28.1%)	19 (27.2%)
35–39 yr	34 (19.5%)	48 (27.6%)	25 (26.1%)	21 (30%)
40–44 yr	17 (9.7%)	20 (11.5%)	6 (6.2%)	14 (20%)
45–50 yr	1 (0.6%)	2 (1.1%)	1 (1%)	1 (1.4%)
Parity:				
Nulliparous (P0)	85 (50%)	70 (40.2%)	47 (49%)	17 (24.3%)
Primiparous (P1)	32 (18.8%)	30 (17.2%)	26 (27.1%)	4 (5.7%)
Multiparous (P≥2)	53 (31.2%)	74 (42.6%)	23 (23.9%)	49 (70%)
Unknown	4 (2.3%)	0 (0%)	0 (0%)	0 (0%)
Cause of ESKD:				
Glomerulonephritis (GN)	42 (24.1%)	41 (23.6%)	23 (24.0%)	17 (24.3%)
Hypertensive nephropathy/Vascular	29 (16.7%)	25 (14.4%)	7 (7.3%)	16 (22.9%)
Diabetic nephropathy (DN)	12 (6.9%)	13 (7.5%)	10 (10.4%)	3 (4.3%)
Lupus nephritis (SLE)	13 (7.5%)	17 (9.7%)	7 (7.3%)	9 (12.8%)
Pyelonephritis (PN)	5 (2.9%)	12 (6.9%)	10 (10.4%)	0 (0%)
Polycystic kidney disease (PKD)	3 (1.7%)	2 (1.2%)	1 (1.1%)	1 (1.4 %)
Other causes	36 (20.7%)	30 (17.2%)	20 (20.8%)	8 (11.4%)
Unknown	34 (19.5%)	34 (19.5%)	18 (18.8%)	16 (22.9%)
Comorbidities and risk factors before pregnancy:				
At least one declared comorbidity, including:	38 (27.7%)	35 (25%)	11 (13.9%)	23 (39.7%)
Diabetes	18 (10.4%)	21 (12.4%)	13 (13.5%)	8 (12.3%)
Lupus	13 (7.5%)	17 (9.7%)	7 (7.3%)	9 (12.9%)
Liver disease (HBV, HCV, cirrhosis)	10 (5.9%)	13 (7.7%)	1 (1.1%)	11 (16.7%)
HIV/AIDS	5 (2.9%)	6 (3.4%)	1 (1.1%)	4 (5.8%)
Obesity (BMI ≥30 kg/m^2^)	12 (7.6%)	24 (15.1%)	13 (15.5%)	9 (13.4%)
Active smoking	23 (14.8%)	18 (11.3%)	10 (11.4%)	7 (10.9%)
Activity:				
Active	51 (29.3%)	36 (20.7%)	22 (22.9%)	10 (14.3%)
Stay-at-home woman	14 (8.1%)	21 (12%)	13 (13.6%)	7 (10%)
Unemployed	2 (1.1%)	4 (2.3%)	3 (3.1%)	0 (0%)
Inactive (long sick leave and other)	40 (23%)	41 (23.6%)	24 (25%)	16 (22.9%)
Unknown	67 (38.5%)	72 (41.4%)	37 (35.44%)	37 (52.8%)
Pregnancy period:				
2006–2010	30 (17.2%)	31 (17.8%)	14 (14.6%)	10 (14.3%)
2011–2015	77 (44.3%)	63 (36.2%)	41 (42.7%)	21 (30%)
2016–2020	67 (38.5%)	80 (46%)	41 (42.7%)	39 (55.7%)
Timing of conception:				
Conception on chronic dialysis (COD)	162 (93.1%)	130 (74.7%)	71 (74%)	54 (77.1%)
Conception before starting dialysis (CBD)	12 (6.9%)	44 (25.3%)	25 (26%)	16 (22.9%)
Dialysis method during trimester 1:	*N* = 174	*N* = 145	*N* = 96	*N* = 70
Hemodialysis (HD)	142 (81.6%)	118 (81.3%)	74 (77.1%)	39 (55.7%)
Peritoneal dialysis (PD)	5 (2.9%)	3 (2.1%)	2 (2.1%)	1 (1.4%)
Unknown	27 (15.5%)	24 (16.5%)	4 (4.1%)	20 (28.6%)
Conception before starting dialysis (CBD)	0 (0%)	29 (16.7%)	16 (16.7%)	10 (14.3%)
Dialysis mode during trimester 1:	*N* = 174	*N*= 145	*N* = 96	*N* = 70
Conventional dialysis	106 (60.9%)	45 (31%)	23 (23.9%)	20 (28.6%)
Daily dialysis	22 (12.6%)	61 (42%)	51 (53.1%)	8 (11.4%)
Unknown	46 (26.5%)	39 (27%)	6 (6.3%)	32 (45.7%)
Conception before starting dialysis (CBD)	0 (0%)	29 (16.7%)	16 (16.7%)	10 (14.3%)
Dialysis method during trimester 2:	*N* = 7 (− 167)	*N* = 165 (− 2 + 22)	*N* = 94 (− 2)	*N* = 70
Hemodialysis (HD)	7 (100%)	126 (76.4%)	85 (90.4%)	34 (48.6%)
Peritoneal dialysis (PD)	0 (0%)	3 (1.8%)	2 (2.1%)	1 (1.4%)
Unknown	0 (0%)	36 (21.8%)	6 (6.3%)	30 (42.9%)
Conception before starting dialysis (CBD)	0 (0%)	7 (4%)	1 (1.1%)	5 (7.1%)
Dialysis mode during trimester 2:	*N* = 7 (− 167)	*N* = 165 (− 2 + 22)	*N* = 94 (− 2)	*N* = 70
Conventional dialysis	1 (14.3%)	13 (7.9%)	7 (7.4%)	6 (8.6%)
Daily dialysis	6 (85.7%)	96 (58.2%)	79 (84.1%)	14 (20%)
Unknown	0 (0%)	56 (33.9%)	7 (7.4%)	45 (64.3%)
Conception before starting dialysis (CBD)	0 (0%)	7 (4%)	1 (1.1%)	5 (7.1%)
Dialysis method during trimester 3:	*N* = 0	*N* = 151 (− 21 + 7)	*N* = 74 (− 20)	*N* = 70
Hemodialysis (HD)		110 (72.9%)	66 (89.2%)	37 (52.9%)
Peritoneal dialysis (PD)		2 (1.3%)	2 (2.7%)	0 (0%)
Unknown		39 (25.8%)	6 (8.1%)	33 (47.1%)
Dialysis mode during trimester 3:	*N* = 0	*N* = 151 (− 21 + 7)	*N* = 74 (− 20)	*N* = 70
Conventional dialysis		2 (1.3%)	1 (1.3%)	1 (1.4%)
Daily dialysis		83 (55%)	62 (83.8%)	18 (25.7%)
Unknown		66 (43.7%)	11 (14.9%)	51 (72.9%)

BMI, body mass index; HBV, hepatitis B virus; HCV, hepatitis C virus; IQR, interquartile range; Wk, weeks.

On December 31, 2020, at the end of this study, out of 239 women alive at the end of their pregnancy, 155 (64.8%) had received a transplant, 71 (29.7%) were still on chronic dialysis, and 13 (5.5%) had died during follow-up, with a median time of 2 years (range 0.2–7.6 years) since the parenting event.

## Discussion

This national study was conducted to estimate the incidence of pregnancies in France among women of childbearing age (15–50 years) receiving chronic dialysis from 2006 to 2020 and to describe the pregnancy outcomes (live birth rate, gestational age, rate of miscarriages, and abortions) and renal management during pregnancy. There were 348 pregnancies identified in 240 women. The overall incidence rate of pregnancy was 11.12, 95% CI: (9.97–12.37) cases per 1000 person-years. This represents a higher rate compared with the Australian and New Zealand registry (ANZDATA) study with a pregnancy rate of 3.3 per 1000 person-years (1996–2008) but a lower rate than that of the United States registry (USRDS) study with a pregnancy rate of 17.7 per 1000 person-years (2005–2013), probably because of the fact that they were from different periods.^2^^,^^3^

Obesity remains a risk factor for multiple complications, such as diabetes, hypertension, preeclampsia, and preterm birth.^17^^,^^18^ In our study, obesity was observed in 12.9% of women whereas in the French general population it was estimated at 14.4 %^19^ In our previous review, the frequency was 34.2%, ranging from 7.1% to 38.7%.^5^

COD was observed in 83.9% of pregnancies, 93.1% of abortions (<22 weeks) and 74.7% of deliveries (≥22 weeks). CBD was observed in 16.1% of pregnancies, with a median (Interquartile range) glomerular filtration rate at initiation of 9 (6-13) ml/min/1.73 m^2^ (CKD-EPI formula) and emergency start in 16 (28.6%) of them.^6^, ^7^, ^8^, ^9^, ^10^, ^11^, ^12^, ^13^ These pregnancies (CBD) resulted in 6.9% of abortions and 25.3% of deliveries (Table 4). Women who had had a pregnancy loss before starting dialysis were not included in this study, even if they began dialysis after that loss. It should be noted that, although CBD resulted in a higher birth rate, this group had a higher proportion of preterm births (26% of deliveries at 22–36 weeks) versus 22.9% deliveries at term (≥37 weeks), whereas in COD there were 74% versus 77.1% respectively, in keeping with the literature.^20^

Despite no difference between maternal and fetal outcomes between HD and PD,^21^^,^^22^ in our study, HD was the predominant modality during pregnancy. Only 2.5% of women were treated with PD at the first trimester, and only 1.3% were still on PD at the third trimester. This is lower than 7.4% found in the literature,^5^ and can be partly explained by the low overall use of PD in France but also fear of complications. In fact, the overdistension of uterus at the end of the pregnancy may have a negative influence on the mother’s nutritional status and may require lower volumes of dialysate. Furthermore, complications such as peritonitis, catheter displacement, drain pain, catheter-related uterine trauma and the hemoperitoneum were described.^3^^,^^21^^,^^23^^,^^24^

In the literature, an intensive dialysis regimen (20–36 h/wk) was associated with a positive correlation on pregnancy outcomes because it facilitates excess fluid removal, allows better control of blood pressure, and increases clearance of uremic toxins. In our study, we only had data on the frequency of weekly dialysis sessions but no information on the length of those sessions. There were 1.4% cases of polyhydramnios in our study, which is very low compared with the 17% found in the literature, and intrauterine growth restriction was present in 4.5% versus 5.9%.^5^^,^^8^^,^^9^^,^^24^, ^25^, ^26^

In our study, the incidence of hypertensive disorders during pregnancy was lower than previously described: preeclampsia (for pregnancies reached ≥22 weeks) 10.3% versus 11.9%, gestational hypertension 2% versus 7.7%, and eclampsia 0.3% versus 0.7%.^5^ In the general population, gestational hypertension develops in 5% to 6% of pregnancies (6–17% in nulliparous women and 2–4% in multiparous women),^18^^,^^27^, ^28^, ^29^ and preeclampsia in 1% to 2% of pregnancies (2–7% in healthy nulliparous women, more in presence of risk factors including obesity, diabetes, age >40 years or <18 years, autoimmune disease, a change of sexual partner, or insufficient exposure to the partner’s sperm i.e., prolonged use of a condom).^26^, ^27^, ^28^, ^29^, ^30^ In our study, the proportion of nulliparous women arriving at 20 weeks of gestation was 69 of 179 (38.5%), a remarkable feat in the management of these pregnancies and probably because of daily dialysis and the growing experience of dialysis centers.

Gestational diabetes was observed in 11 women (3.1%), representing a higher rate than described in a previous review (0.5%).^5^ This is consistent with the fact that the frequency of gestational diabetes in pregnant women has increased in the general French population from 10.8% in 2016 to 16.4% in 2021. This is probably partly explained by the increase in the frequency of screening tests but also the rise in prevalence of risk factors (maternal age and obesity) or by increasing consideration of the diagnostic criteria appearing in the recommendations for clinical practice since 2010.^17^^,^^31^^,^^32^

There are 2 types of termination of pregnancy, therapeutic abortion performed for reasons of maternal health or fetal disease, and elective abortion performed because a woman has chosen to end her pregnancy. Our study shows a significant proportion (29.9%) of elective abortions, the highest proportion was of patients belonging to the age group between 30 and 34 years (30.8%), diabetics (9.6%), smokers (11.5%), active women (28.8%), in HD (75%), and mainland France as well as overseas territories (Supplementary Table S2 and Supplementary Figure S2). In the general French population with the proportion of 15.5 per 1000 women aged between 15 and 49 years (14.9 per 1000 in metropolitan France and 29.5 in overseas territories). This is probably explained by the patient’s fear and the medical team’s reluctance toward their pregnancies. It may also be explained by a possible recourse to elective abortion rather than therapeutic abortion, a more straightforward procedure. Our study suggests that physicians should counsel women of childbearing age in chronic dialysis on the possibility of contraception if a pregnancy is not wanted. An unwanted pregnancy can have a psychological and clinical impact on these patients, such as the risk of hemorrhage, infection, failed abortion, and the side effects of different treatments.^4^^,^^33^

In our study, 174 deliveries (≥22 weeks) ended with 166 live births, i.e., 47.7% of the 348 pregnancies. In the literature, the live birth rate represented 71.4%^5^ but most studies did not include elective abortions.

There was a lower stillbirth rate in our study than in the literature (4.5% vs. 8.3%).^5^ This may be due to improvements in dialysis conditions, adequate fetal monitoring, progress in obstetrics and neonatal care, and the greater knowledge of the mechanisms of complications, lead to better prevention.^4^^,^^25^

In kidney transplantation, good short-term pregnancy as well as graft outcomes is observed.^34^ However, given the recent improvement in the prognosis of patients with HD, it should no longer curb the desire for pregnancy in these patients. Further studies are needed to discuss the right time to start a pregnancy for women on dialysis who are waiting for a kidney transplant.^35^ A balance must be struck between the unpredictable waiting time for a transplant and the patient’s increasing age, which reduces their chances of conceiving.

### Strengths and Limitations

The strong point of our study is the representativeness of the 2 national databases used. REIN is a national registry of recognized quality that contains epidemiological information on kidney health, and the SNDS contains information on the consumption of medical care for almost the entire French population. Secondly, we used objective care indicators recorded in the SNDS, which spared us from any possible memory or information biases. Thirdly, our study covered all pregnancy outcomes (spontaneous miscarriages, abortions, ectopic pregnancies, and births).

However, when interpreting our study, the following limitations should be borne in mind: it only concerns abortions recorded in hospitals, some patients may have had early miscarriages treated by their general practitioner, although this is unlikely in women who underwent dialysis. This would result in an underestimation of the pregnancy incidence rate. Secondly, because of the lack of information in the SNDS concerning the duration of dialysis sessions and biochemical and clinical data during pregnancy, this could not be analyzed. Thirdly, some coding errors or under-reporting, inherent to such big hospital claims database may have occurred that could explain lower complications like preeclampsia. Finally, in France we do not have access to ethnicity because women of Black and Asian ethnicity may be at increased risk of miscarriage when compared with Caucasian women.^36^

## Conclusion

The incidence of pregnant women receiving chronic dialysis in France seems to have become stable in recent years. There have been improvements in maternal and fetal outcomes compared with the literature. However, our study shows a significant proportion of elective abortions. Physicians should counsel women of childbearing age receiving chronic dialysis about the possibility of contraception if pregnancy is not desired. Further studies are needed to confirm the results of our study. Finally, we could benefit from continuous registration of pregnancies in women receiving chronic dialysis to obtain more data to identify specific predictors that may influence the outcome of these pregnancies.

## Disclosure

BH and JJ-P have received research grants from the French Agency of Biomedicine that is a public institution. All the authors declared no competing interests.

## Data Sharing Statement

All data used for this study were extracted from the French National Health Data System (SNDS) and the French ESKR REIN registry. The data cannot be made publicly available due to legal restrictions. Aggregated data are available upon request following some procedures. More information regarding data access to SNDS can be found at: https://documentation-snds.health-data-hub.fr/snds/formation_snds/documents_cnam/guides_pedagogiques_snds/guide_pedagogique_acpermanents.html#qui-a-acces-au-snds-et-a-quelles-donnees. For the access to REIN data, the contact person is Cécile Couchoud at the Agence de la biomédecine: cecile.couchoud@biomedecine.fr**.**

## Supplementary Material

Supplementary File (PDF) **Figure S1.** Flowchart of the study population for the period 2006–2020. **Figure S2.** Distribution of pregnancies on chronic dialysis and their outcomes in mainland France and overseas departments and territories between 2006 and 2020. **Table S1.** ICD-10 diagnoses, CCAM acts, and GHM codes. **Table S2.** Characteristics of pregnancies resulted in abortions. (<22 weeks). **STROBE Statement**.

## Footnotes

Supplementary File (PDF)
**Supplementary Figure S1.** Flowchart of the study population for the period 2006–2020. **Supplementary Figure S2**. Distribution of pregnancies on chronic dialysis and their outcomes in mainland France and overseas departments and territories between 2006 and 2020. **Supplementary Table S1.** ICD-10 diagnoses, CCAM acts, and GHM codes. **Supplementary Table S2.** Characteristics of pregnancies resulted in abortions (>22 weeks).
**STROBE Statement.**
