# Supplementary material for: Pregnancy Among Women Receiving Chronic Dialysis in France (2006–2020)
Source: Kidney Int Rep. 2024 May 15;9(8):2432–42. doi: 10.1016/j.ekir.2024.05.008 (PMC11328538; doi:10.1016/j.ekir.2024.05.008)
Supplement: Supplementary File (PDF) — Figure S1. Flowchart of the study population for the period 2006–2020. Figure S2. Distribution of pregnancies on chronic dialysis and their outcomes in mainland France and overseas departments and territories between 2006 and 2020. Table S1. ICD-10 diagnoses, CCAM acts, and GHM codes. Table S2. Characteristics of pregnancies resulted in abortions. (<22 weeks). STROBE Statement. [file mmc1.pdf]

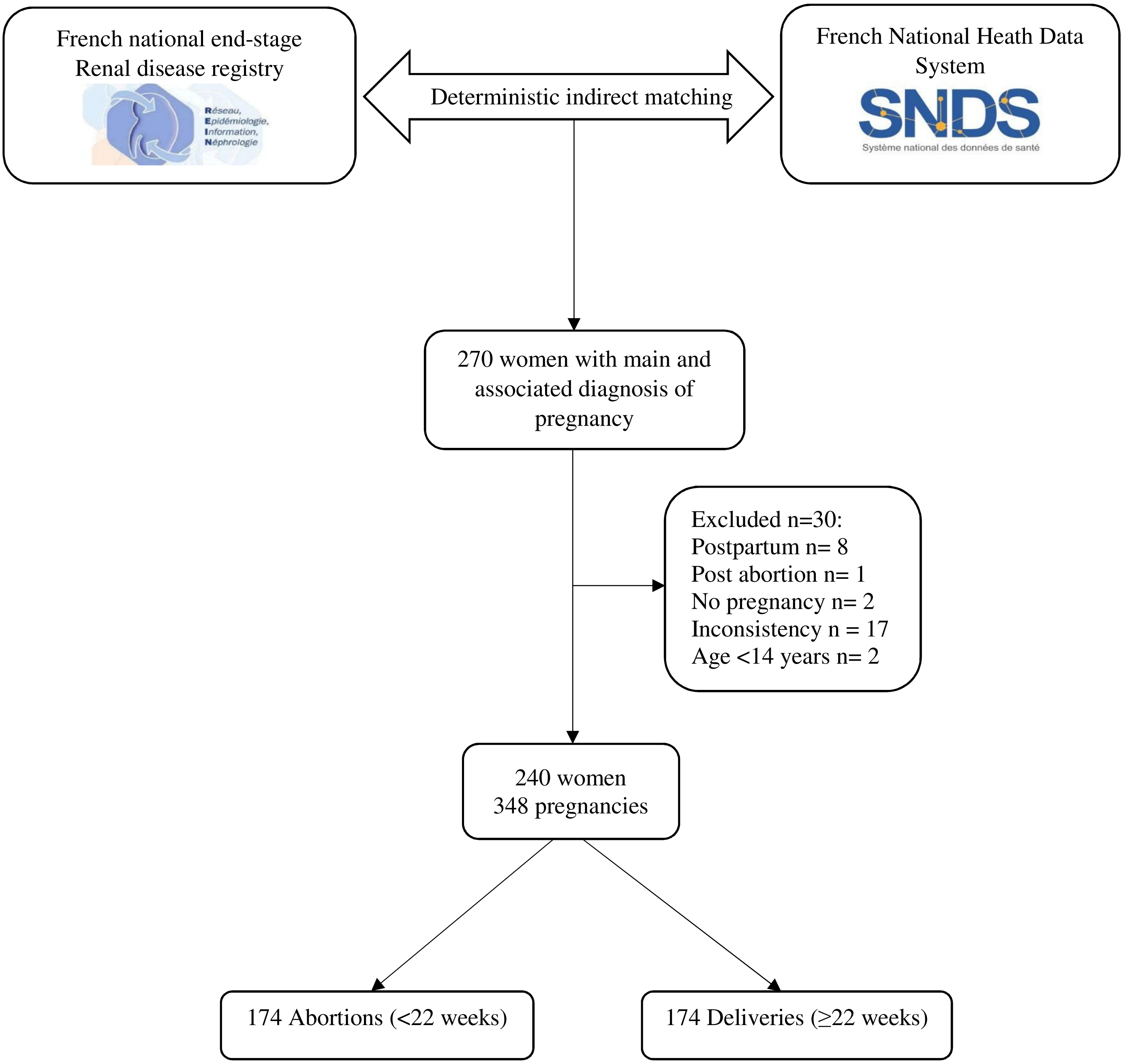

Supplementary Figure 1. Flow chart of the study population for the period 2006–2020

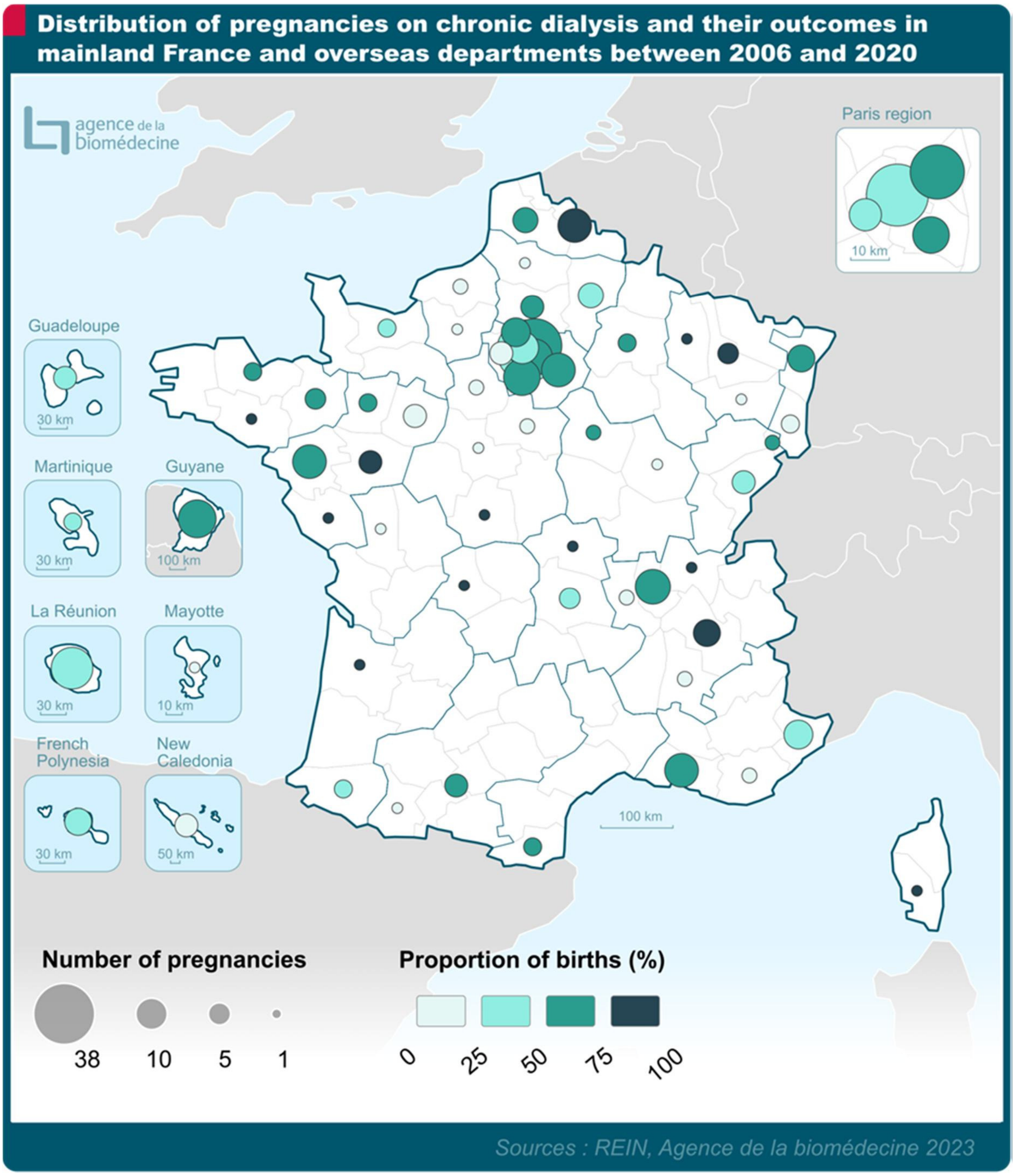

Supplementary Figure 2. Distribution of pregnancies on chronic dialysis and their outcomes in mainland France and overseas departments and territories between 2006 and 2020.

Supplementary Table 1. ICD-10 diagnoses, CCAM acts, and GHM codes

Variable	ICD-10 diagnoses	CCAM acts	GHM codes
**Maternal complications (pregnancy)**			
Gestational hypertension	O13		
Pre-eclampsia	O11, O140, O141, O149		
Eclampsia	O152		
Gestational Diabetes	O244		
Liver disease during pregnancy	O266		
Postpartum hemorrhage	O720		
Anemia	O990		
Amniotic embolism	O881		
Maternal death	O990, A410, Z358		18M13E
**Obstetric complications**			
Preterm spontaneous labor	O600, O601		
Premature rupture of membranes	O420, O421, O756		
Polyhydramnios	O40		
Abruption placenta	O458, O459		
Another hemorrhage before labor	O468		
**Total abortion** (<22 weeks)			
Elective abortion	O048, O049, O0490, Z640	JNJD002, JNJP001, JNMD001	
Spontaneous abortion (miscarriages)	O031, O034, O036, O038, O039	JNJD001, JNJD002, JNJP001, JNMD001	
Therapeutic abortion	O041, O044, O046, O048, O049, O100, O102, O281, O288, O289, O998	JNJD001, JNJD002, JNJP001, JNMD001	
Ectopic pregnancy	O001, O008	JJFC001, JJPC001	
Hydatidiform mole	O020, O028	JNJD002 JNJP001	
**Total childbirth** (≥22 weeks)			
Childbirths	O300, O601, O603, O800, O814, O820, O821	JNJD001, JQGA002, JQGA003, JQGA004, JQGD001, JQGD002, JQGD004, JQGD007, JQGD010, JQGD012, JQGD013	14C02A,14C02B, 14C02C, 14C06A, 14C07D, 14C08A, 14C08B, 14C08C, 14C08D, 14Z02A, 14Z02B,14Z02C, 14Z02T, 14Z11B, 14Z12A, 14Z13A, 14Z13B, 14Z13C, 14Z13D,14Z14A, 14Z14B, 14Z14C, 14Z14D, 14Z14T
Live births	Z370	JQGA002, JQGA003, JQGA004, JQGD001, JQGD002, JQGD004, JQGD007, JQGD010, JQGD012	14C02A,14C02B, 14C02C, 14C06A, 14C07D, 14C08A, 14C08B, 14C08C, 14C08D, 14Z02A, 14Z02B,14Z02C, 14Z02T, 14Z11B, 14Z12A, 14Z13A, 14Z13B, 14Z13C, 14Z13D,14Z14A, 14Z14B, 14Z14C, 14Z14D, 14Z14T
Stillbirth	O363, O364, O468, Z371		14Z10A, 14Z10B, 14Z10T

**Fetal and neonatal complications**			
Fetal heart rate abnormalities	O680, O682		
Intrauterine growth retardation	O365		
Hypoxia / fetal distress	O363, O683		

ICD: International Classification of Diseases

CCAM: Common Classification of Medical Procedures

GHM: Homogeneous Groups of Patients

Supplementary Table 2. Characteristics of pregnancies resulted in abortions (<22 weeks)

Characteristics	Abortions (<22 weeks) N=174		
	**Elective abortions N=104**	**Therapeutic abortions N=17**	**Other** (Miscarriages+ectopics+moles) **N=53**
	**N(%), mean ± standard deviation**		
Age at conception (years)	30.6±7.3	31.9±4.8	32.1±5.5
**Age classes:**			
15-19 years	7 (6.7%)	0 (0%)	2 (3.8%)
20-24 years	15 (14.4%)	2 (11.8%)	4 (7.5%)
25-29 years	20 (19.2%)	3 (17.6%)	9 (17%)
30-34 years	32 (30.8%)	7 (41.2%)	21 (39.6%)
35-39 years	18 (17.3%)	4 (23.5%)	12 (22.6%)
40-44 years	11 (10.6%)	1 (5.9%)	5 (9.4%)
45-50 years	1 (1%)	0 (0%)	0 (0%)
**Parity:**			
Nulliparous (P=0)	41 (39.4%)	9 (52.9%)	35 (66%)
Primiparous (P=1)	21 (20.2%)	3 (17.6%)	8 (15.1%)
Multiparous (P≥2)	41 (39.4%)	5 (29.4%)	7 (13.2%)
Unknown	1 1%)	0 (0%)	3 (5.7%)
**Cause of ESKD:**			
Glomerulonephritis (GN)	23 (22.1%)	8 (47.1%)	11 (20.8%)
Hypertensive nephropathy/Vascular	19 (18.3%)	0 (0%)	10 (18.9%)
Diabetic nephropathy (DN)	5 (4.8%)	0 (0%)	7 (13.2%)
Lupus nephritis (SLE)	8 (7.7%)	2 (11.8%)	3 (5.7%)
Pyelonephritis (PN)	5 (4.8%)	0 (0%)	0 (0%)
Polycystic kidney disease (PKD)	2 (1.9%)	0 (0%)	1 (1.9%)
Other causes	21 (20.2%)	4 (23.5%)	11 (20.8%)
Unknown	21 (20.2%)	3 (17.6%)	10 (18.9%)
**Comorbidities and risk factors:**			
At least one declared comorbidity including:	21 (20.2%)	4 (23.5%)	13 (24.5%)
Diabetes	10 (9.6%)	0 (0%)	8 (15.1%)
Lupus	8 (7.7%)	2 (11.8%)	3 (5.7%)
Liver disease (HBV, HCV, cirrhosis)	7 (6.7%)	2 (11.8%)	1 (1.9%)
HIV/AIDS	2 (1.9%)	0 (0%)	3 (5.7%)
Obesity (BMI ≥30 kg/m²)	4 (3.8%)	1 (5.9%)	7 (13.2%)
Active smoking	12 (11.5%)	1 (5.9%)	10 (18.9%)
**Activity:**			
Active	30 (28.8%)	4 (23.5%)	17 (32.1%)
Stay-at-home woman	8 (7.7%)	2 (11.8%)	4 (7.5%)
Unemployed	1 (1%)	0 (0%)	1 (1.9%)
Inactive (long sick leave and other)	19 (18.3%)	6 (35.3%)	15 (28.3%)
Unknown	46 (44.2%)	5 (29.4%)	16 (30.2%)
**Pregnancy period:**			

2006-2010	15 (14.4%)	5 (29.4%)	10 (18.9%)
2011-2015	49 (47.1%)	6 (35.3%)	22 (41.5%)
2016-2020	40 (38.5%)	6 (35.3%)	21 (39.6%)
**Timing of conception:**			
Conception on dialysis (COD)	99 (95.2%)	16 (94.1%)	47 (88.7%)
Conception before starting dialysis (CBD)	5 (4.8%)	1 (5.9%)	6 (11.3%)
**Dialysis method (Trimester 1)**			
Hemodialysis	78 (75%)	17 (100%)	47 (88.7%)
Peritoneal dialysis	4 (3.8%)	0 (0%)	1 (1.9%)
Unknown	22 (21.2%)	0 (0%)	5 (9.4%)
**Registration on the waiting list at conception:**			
Conception on dialysis (COD)	45 (43.26%)	11 (64.7%)	22 (41.5%)
Conception before starting dialysis (CBD)	0 (0%)	1 (5.8%)	3 (5.6%)
**Residence location :**			
Metropolitan France	84 (80.8%)	15 (88.2%)	43 (81.1%)
Overseas territories	20 (19.2%)	2 (11.8%)	10 (18.9%)

HIV: human immunodeficiency virus, AIDS: acquired immuno deficiency syndrome, HBV: hepatitis B virus, HCV: hepatitis C virus, BMI: Body Mass Index.

STROBE Statement—checklist of items that should be included in reports of observational studies

	Item No.	Recommendation	Page No.	Relevant text from manuscript
**Title and abstract**	1	(*a*) Indicate the study's design with a commonly used term in the title or the abstract	1	
		(*b*) Provide in the abstract an informative and balanced summary of what was done and what was found	2	
		**Introduction**		
Background/rationale	2	Explain the scientific background and rationale for the investigation being reported	3	
Objectives	3	State specific objectives, including any prespecified hypotheses	4	
		**Methods**		
Study design	4	Present key elements of study design early in the paper	4	
Setting	5	Describe the setting, locations, and relevant dates, including periods of recruitment, exposure, follow-up, and data collection	4, 5	
Participants	6	(*a*) *Cohort study*—Give the eligibility criteria, and the sources and methods of selection of participants. Describe methods of follow-up<br>*Case-control study*—Give the eligibility criteria, and the sources and methods of case ascertainment and control selection. Give the rationale for the choice of cases and controls<br>*Cross-sectional study*—Give the eligibility criteria, and the sources and methods of selection of participants	4	
		(*b*) *Cohort study*—For matched studies, give matching criteria and number of exposed and unexposed<br>*Case-control study*—For matched studies, give matching criteria and the number of controls per case	4	
Variables	7	Clearly define all outcomes, exposures, predictors, potential confounders, and effect modifiers. Give diagnostic criteria, if applicable	5	
Data sources/ measurement	8*	For each variable of interest, give sources of data and details of methods of assessment (measurement). Describe comparability of assessment methods if there is more than one group	*5*	
Bias	9	Describe any efforts to address potential sources of bias	11	
Study size	10	Explain how the study size was arrived at	4, 5	

Continued on next page

Quantitative variables	11	Explain how quantitative variables were handled in the analyses. If applicable, describe which groupings were chosen and why	5	
Statistical methods	12	(*a*) Describe all statistical methods, including those used to control for confounding	5	
		(*b*) Describe any methods used to examine subgroups and interactions	6	
		(*c*) Explain how missing data were addressed		
		(*d*) *Cohort study*—If applicable, explain how loss to follow-up was addressed<br>*Case-control study*—If applicable, explain how matching of cases and controls was addressed<br>*Cross-sectional study*—If applicable, describe analytical methods taking account of sampling strategy		
		(*e*) Describe any sensitivity analyses		
**Results**				
Participants	13*	(a) Report numbers of individuals at each stage of study—eg numbers potentially eligible, examined for eligibility, confirmed eligible, included in the study, completing follow-up, and analysed	6	
		(b) Give reasons for non-participation at each stage		
		(c) Consider use of a flow diagram	25	Supplementary Figure S1
Descriptive data	14*	(a) Give characteristics of study participants (eg demographic, clinical, social) and information on exposures and potential confounders	7	Table 2
		(b) Indicate number of participants with missing data for each variable of interest		Table 2
		(c) *Cohort study*—Summarise follow-up time (eg, average and total amount)		
Outcome data	15*	*Cohort study*—Report numbers of outcome events or summary measures over time	*7, 8*	*Table 3*
		Case-control study—Report numbers in each exposure category, or summary measures of exposure		
		Cross-sectional study—Report numbers of outcome events or summary measures		
Main results	16	(*a*) Give unadjusted estimates and, if applicable, confounder-adjusted estimates and their precision (eg, 95% confidence interval). Make clear which confounders were adjusted for and why they were included	8	Table 5
		(*b*) Report category boundaries when continuous variables were categorized		
		(*c*) If relevant, consider translating estimates of relative risk into absolute risk for a meaningful time period		

Continued on next page

Other analyses	17	Report other analyses done—eg analyses of subgroups and interactions, and sensitivity analyses	8, 28	Table 4 and Supplementary Table S1.
**Discussion**				
Key results	18	Summarise key results with reference to study objectives	8, 9, 10, 11	
Limitations	19	Discuss limitations of the study, taking into account sources of potential bias or imprecision. Discuss both direction and magnitude of any potential bias	11	
Interpretation	20	Give a cautious overall interpretation of results considering objectives, limitations, multiplicity of analyses, results from similar studies, and other relevant evidence	11	
Generalisability	21	Discuss the generalisability (external validity) of the study results	11	
**Other information**				
Funding	22	Give the source of funding and the role of the funders for the present study and, if applicable, for the original study on which the present article is based	12	

*Give information separately for cases and controls in case-control studies and, if applicable, for exposed and unexposed groups in cohort and cross-sectional studies.

Note: An Explanation and Elaboration article discusses each checklist item and gives methodological background and published examples of transparent reporting. The STROBE checklist is best used in conjunction with this article (freely available on the Web sites of PLoS Medicine at http://www.plosmedicine.org/, Annals of Internal Medicine at http://www.annals.org/, and Epidemiology at http://www.epidem.com/). Information on the STROBE Initiative is available at www.strobe-statement.org.
